# Autophagic pathway contributes to low-nitrogen tolerance by optimizing nitrogen uptake and utilization in tomato

**DOI:** 10.1093/hr/uhac068

**Published:** 2022-03-23

**Authors:** Jiajian Cao, Xuelian Zheng, Dongling Xie, Hui Zhou, Shujun Shao, Jie Zhou

**Affiliations:** College of Horticulture, Hunan Agricultural University, Nonda Road 1, Changsha, 410128, China; Department of Horticulture/Zhejiang Provincial Key Laboratory of Horticultural Plant Integrative Biology, Zhejiang University, Yuhangtang Road 866, Hangzhou, 310058, China; Department of Horticulture/Zhejiang Provincial Key Laboratory of Horticultural Plant Integrative Biology, Zhejiang University, Yuhangtang Road 866, Hangzhou, 310058, China; Department of Horticulture/Zhejiang Provincial Key Laboratory of Horticultural Plant Integrative Biology, Zhejiang University, Yuhangtang Road 866, Hangzhou, 310058, China; Department of Horticulture/Zhejiang Provincial Key Laboratory of Horticultural Plant Integrative Biology, Zhejiang University, Yuhangtang Road 866, Hangzhou, 310058, China; Department of Horticulture/Zhejiang Provincial Key Laboratory of Horticultural Plant Integrative Biology, Zhejiang University, Yuhangtang Road 866, Hangzhou, 310058, China; Key Laboratory of Horticultural Plants Growth, Development and Quality Improvement, Agricultural Ministry of China, Yuhangtang Road 866, Hangzhou, 310058, China; Department of Horticulture/Zhejiang Provincial Key Laboratory of Horticultural Plant Integrative Biology, Zhejiang University, Yuhangtang Road 866, Hangzhou, 310058, China; Key Laboratory of Horticultural Plants Growth, Development and Quality Improvement, Agricultural Ministry of China, Yuhangtang Road 866, Hangzhou, 310058, China

## Abstract

Autophagy is a primary process involved in the degradation and reuse of redundant or damaged cytoplasmic components in eukaryotes. Autophagy has been demonstrated to facilitate nutrient recycling and remobilization by delivering intracellular materials to the vacuole for degradation in plants under nutrient starvation. However, the role of autophagy in nitrogen (N) uptake and utilization remains unknown. Here, we report that the ATG6-dependent autophagic pathway regulates N utilization in tomato (*Solanum lycopersicum*) under low-nitrogen (LN) conditions. Autophagy-disrupted mutants exhibited weakened biomass production and N accumulation compared with wild-type (WT), while *ATG6* overexpression promoted autophagy and biomass production under LN stress. The N content in *atg6* mutants decreased while that in *ATG6*-overexpressing lines increased due to the control of N transporter gene expression in roots under LN conditions. Furthermore, ATG6*-*dependent autophagy enhanced N assimilation efficiency and protein production in leaves. Nitrate reductase and nitrite reductase activities and expression were compromised in *atg6* mutants but were enhanced in *ATG6*-overexpressing plants under LN stress. Moreover, ATG6-dependent autophagy increased plant carbon fixation and photosynthetic capacity. The quantum yield of photosystem II, photosynthetic N use efficiency and photosynthetic protein accumulation were compromised in *atg6* mutants but were restored in *ATG6*-overexpressing plants. A WT scion grafted onto *atg6* mutant rootstock and an *atg6* scion grafted onto WT rootstock both exhibited inhibited LN-induced autophagy and N uptake and utilization. Thus, ATG6-dependent autophagy regulates not only N uptake and utilization as well as carbon assimilation but also nutrient recycling and remobilization in tomato plants experiencing LN stress.

## Introduction

Plants require multiple nutrients to achieve robust growth and development. Nitrogen (N) is one of the primary macronutrients required by plants, and ~60% of the annual fertilizer consumed worldwide is N fertilizer [1, 2]. N deficiency affects crop yield and quality and is thus recognized as a widespread problem worldwide [3]. In Africa, N deficiency threatens 80% of crop production and causes low crop yields, food insecurity and malnutrition [3, 4]. Therefore, how to improve N utilization under N-limited conditions is our research focus.

N deficiency first affects the N uptake and assimilation systems in plants [1]. Nitrate, the main source of N for plants, is absorbed and transported by nitrate transporters, including those of the nitrate transporter 1/peptide transporter (NRT1/PTR) family and the NRT2 family [1]. In response to different nitrate conditions, plants have evolved two classes of influx transporters; one is the high-affinity transport system, which functions mainly in low-nitrate conditions (<1 mM NO_3_^−^), and the other is the low-affinity transport system, which functions mainly in high-nitrate conditions (>1 mM NO_3_^−^) [5]. Most isoforms of NRT1 family members show low nitrate affinity, although NRT1.1 has been proven to be the only dual-affinity nitrate transporter so far [1]. Most NRT2 family members function as high-affinity nitrate transporters in the roots, and their interaction facilitates efficient soil nitrate utilization at low nitrate concentrations [6]. For example, knockout of *NRT2.1* resulted in the loss of up to 72% of the root NO_3_^−^ influx in *Arabidopsis* [7]. Nitrate can be metabolized directly in the roots after absorption and stored in the vacuole, but most nitrate is transported to aerial parts of the plants. Nitrate is initially converted to nitrite (NO_2_^−^) by nitrate reductase (NR) in the cytoplasm and then to ammonium (NH_4_^+^) in the plastids by nitrite reductase (NiR). Ammonium is further transformed into amino acids via the glutamine synthetase (GS)/glutamine-2-oxoglutarate aminotransferase (GOGAT) cycle [2]. Under N deficiency, plants exhibited a significant decrease in the transcript and activity levels of NR, NiR, GS, and GOGAT [8]. The overexpression of the genes for these N transporter and assimilation enzymes improves N use efficiency (NUE). For instance, the overexpression of *OsNRT2.3b* enhanced N uptake and improved yield under both high- and low-N conditions in rice [9], and the overexpression of *ZmGS1.3* also increased grain production in maize [10].

N and carbon (C) metabolism are tightly interrelated in plants [11]. N deficiency affects not only N uptake and assimilation but also C assimilation, especially photosynthesis. Photosynthesis, and subsequently respiration, provides the C skeletons and energy required for the synthesis of amino acids and proteins [12]. N in the leaves is used mainly to synthesize photosynthesis-related proteins for C assimilation and metabolism; for example, 12–35% of leaf N is contained in ribulose-1,5-bisphosphate (RuBP) carboxylase/oxygenase (Rubisco), the major enzyme involved in C fixation [13]. Under low-nitrogen (LN) conditions, an LN-tolerant genotype of wheat had higher Rubisco activation and photosynthesis than an LN-sensitive genotype [14]. Comparative genome and transcriptome analyses also revealed that photosynthesis genes were more abundant in a *Brassica napus* N-efficient genotype than in an N-inefficient genotype under N starvation [15].

Protein degradation for N recycling helps plants adapt to N deficiency. Autophagy is one of the most important degradation and recycling pathways for proteins and cytoplasmic organelles, and it plays critical roles in nutrient recycling and remobilization under nutrient starvation [16]. Under N-restricted conditions, Rubisco can be transferred to the vacuole and degraded through an autophagy-related (ATG) gene-dependent autophagic process [17]. Downregulation or mutation of *ATG* genes increases the sensitivity of plants to LN stress. For example, an *Arabidopsis atg9* mutant exhibited chlorosis in cotyledons and rosette leaves and a reduction in seed set under N-starvation conditions [18]. Furthermore, autophagy is also involved in N remobilization in plants. *Arabidopsis atg* mutants were unable to digest or recycle proteins and other N resources in vegetative tissues, causing defects in N remobilization; thus, N partitioning in seeds was significantly lower than that in wild-type (WT) plants under LN conditions [19]. The lack of N severely hindered seedling growth, aggravated leaf senescence, and decreased the NUE of seeds in maize *atg12* mutants [20]. In addition, apple *MdATG18a* overexpression upregulated the transcript levels of *MdNIA2* and *MdNRT2.1*/*2.4*/*2.5* and enhanced leaf nitrate content under LN stress [21]. Recently, we reported that brassinazole resistant 1 (BZR1), one of the important transcription factors downstream of brassinosteroid, accelerated the degradation of insoluble ubiquitinated protein aggregates and increased tolerance to N starvation through the transcriptional regulation of *ATG2* and *ATG6* expression and the induction of autophagy in tomato plants [22]. Although the function of autophagy in internal N recycling has been extensively studied, how autophagy influences N uptake and assimilation and C assimilation processes remains to be investigated. In these experiments, we attempted to use *ATG6*-related tomato materials to further investigate the exact function of autophagy in N uptake and assimilation. ATG6 (VPS30/ATG6 in yeast, BECN1/Beclin1 in mammals) is the core subunit of the class III phosphatidylinositol 3-kinase (PI3K) complex, which is important for phagophore decoration due to its recruitment of other effector proteins [23]. Previous reports on ATG6 were focused on the model plant *Arabidopsis* and show that this protein participates in multiple processes, such as plant pollen development and pathogen immunity [24, 25]. However, few ATG6-related functions in N nutrition management have been reported in vegetables.

In this work, we analyzed the biological function of autophagy under N-limited conditions. We demonstrated that ATG6-dependent autophagy promoted N uptake by regulating the expression of *NRT1.1* and *NRT2.1* in roots and enhanced N assimilation by promoting the activities of NR and NiR under LN conditions. In addition, ATG6-dependent autophagy alleviated the damage induced by LN stress on photosynthesis and plant growth by increasing the accumulation of proteins associated with photosynthesis and photosystem II (PSII) protection. Together, these results provide new information that indicates that autophagy is essential for N uptake and assimilation in conjunction with C assimilation, in addition to its established roles in nutrient recycling and remobilization, under LN stress.

**Figure 1:**
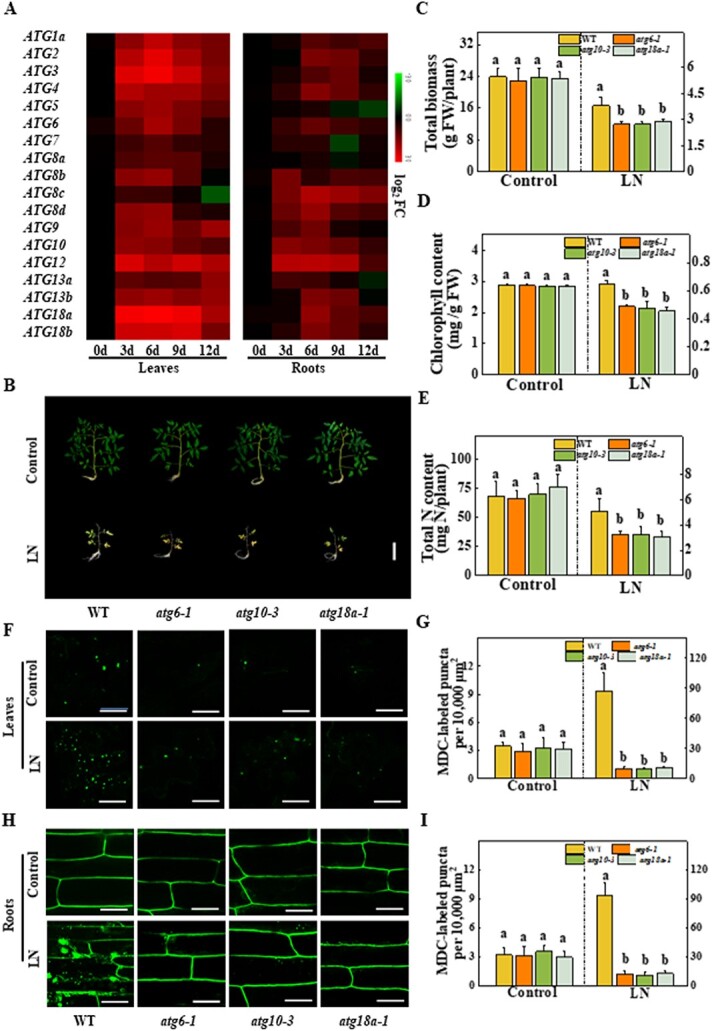
Role of autophagy in the response to LN stress in tomato. **a** Heat map showing the expression of *ATG* genes at different time points after LN stress. qRT–PCR results with three biological replicates were employed to determine transcript levels. MeV version 4.9 was used for cluster analysis, and data were transformed by log_2_-fold change (FC). The color bar on the right shows the levels of expression; 0 days (d), 3 d, 6 d, 9 d and 12 d indicate the time after LN treatment. **b** Phenotypes of WT and *atg* mutants in response to LN stress. Four-week-old plants were transplanted to LN solution and maintained for another 3 weeks. Scale bars = 10 cm. The total biomass of the whole plant (**c**) and the chlorophyll content of the fourth expanded leaf (**d**) were determined on the 21st day under LN stress. **e** Total N content of the whole plant. Data represent the mean of three biological replicates (± standard deviation). MDC-stained autophagosomes were detected in the leaves (**f**) and roots (**h**) of WT, *atg6-1*, *atg10-3*, and *atg18a-1* plants on the fifth day under LN stress by confocal microscopy. MDC-stained autophagosomes are shown as green signals. Scale bars = 25 μm. Autophagic activity was calculated as the number of MDC-labeled puncta per 10 000 μm^2^ in the leaves (**g**) and roots (**i**). For each treatment, >20 pictures were utilized for quantification. All studies were performed three times and similar findings were obtained each time. Different lower-case letters represent significant differences at *P* < .05 according to Tukey’s test. FC, fold change; FW, fresh weight.

## Results

### Tomato autophagy mutants are hypersensitive to low-nitrogen stress

To investigate the temporal expression patterns of *ATG* genes in tomato roots and leaves under LN stress, we first investigated the transcript levels of 18 tomato *ATG* genes at different time points after LN treatment. The results of quantitative real-time PCR (qRT–PCR) showed that a majority of tomato *ATG* genes were upregulated on the 3rd day and remained highly expressed until the 12th day in both roots and leaves after LN treatment (Fig. 1a). To comprehensively understand the effects of autophagy on N starvation, we chose ATG6 (PI3K complex, autophagy initiation), ATG10 (ATG8/12 conjugation systems, autophagosome maturation), ATG18a (ATG9 transmembrane complex, phagophore expansion), which are involved in different autophagy processes and were highly induced by N stress, for relevant verification. We constructed tomato *atg6*, *atg10*, and *atg18a* homozygous mutants using the CRISPR/Cas9 system. Four homozygous gene editing lines, *atg6-1* (2-bp deletion), *atg6-8* (1-bp insertion), *atg10-3* (1-bp insertion) and *atg18a-1* (2-bp insertion), were obtained, and these mutants generated premature stop codons resulting in truncated proteins (Supplementary Data Fig. S1). These *atg* mutants displayed hypersensitivity to N deficiency by exhibiting increased leaf chlorosis and decreased plant biomass and chlorophyll content compared with WT seedlings (Fig. 1b–d). Interestingly, the N contents of the WT and *atg* mutants were similar under N-sufficient (control) conditions; however, the N content was 36.8, 36.0, and 40.2% lower in *atg6*, *atg10*, and *atg18a* seedlings, respectively, than in WT seedlings after 3 weeks of LN conditions (Fig. 1e). Next, we assessed autophagy with monodansylcadaverine (MDC) staining, which involves the fluorescent labeling of acidic vesicles, primarily autophagosomes, and counting of the labeled puncta [26]. Compared with the stronger induction of autophagy in WT leaves and roots, autophagic activity was inhibited by MDC staining in the *atg6-1*, *atg10-3*, and *atg18a-1* mutants under LN stress (Fig. 1f–i). To verify these findings, green fluorescent protein (GFP)-tagged ATG8f (GFP-ATG8f) was employed as a marker to evaluate the formation of autophagosomes during LN stress [27]. As expected, the GFP-ATG8f-labeling results for the *atg6-1*, *atg10-3*, and *atg18a-1* mutants revealed much lower autophagic activity than that in the WT seedlings under LN stress (Supplementary Data Fig. S2A and B). Western blotting analysis further showed that the accumulation of free GFP was inhibited in *atg* mutants under LN stress (Supplementary Data Fig. S2C and D). These results suggest that autophagy is essential for N uptake and seedling growth in tomato under LN stress.

### ATG6 plays a crucial role in low-nitrogen stress

**Figure 2:**
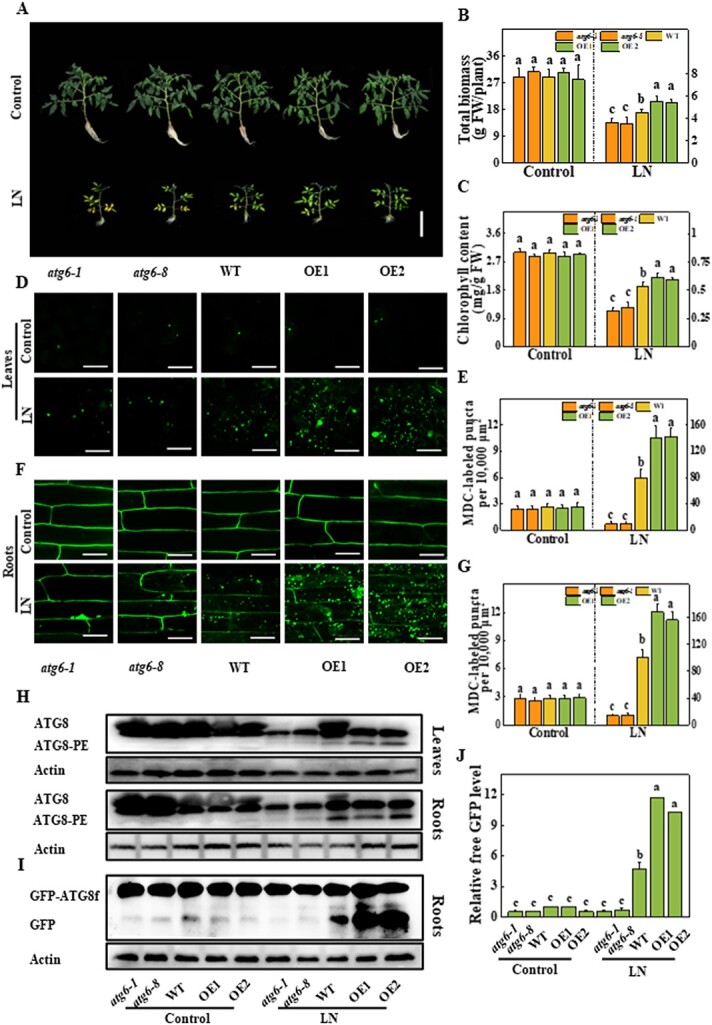
Overexpressing *ATG6* increases LN tolerance. **a** Phenotypes of *atg6-1*, *atg6-8*, WT, and two *ATG6*-overexpressing lines (OE1 and OE2) in response to LN stress. Four-week-old plants were transplanted to LN solution for 3 weeks. Scale bars = 10 cm. The total biomass of the whole plant (**b**) and the chlorophyll content of the fourth expanded leaf (**c**) were determined on the 21st day under LN stress. MDC-stained autophagosomes were detected in the leaves (**d**) and roots (**f**) of *atg6-1*, *atg6-8*, WT, OE1, and OE2 plants on the fifth day under LN stress by confocal microscopy. MDC-stained autophagosomes are shown as green signals. Scale bars = 25 μm. Autophagic activity was calculated as the number of MDC-labeled puncta per 10 000 μm^2^ in the leaves (**e**) and roots (**g**). For each treatment, >20 pictures were utilized for quantification. **h** ATG8 protein levels in the leaves and roots of *atg6-1*, *atg6-8*, WT, OE1, and OE2 plants on the fifth day of LN treatment. ATG8 (non-lipidated form) and ATG8-PE (lipidated form) are indicated on the left. (**i**) Accumulation of GFP-ATG8f proteins in the *GFP-ATG8f-*overexpressing roots of *atg61*, *atg6-8*, WT, OE1, and OE2 plants on the fifth day under LN stress. GFP-ATG8f fusion and free GFP positions are marked on the left. (**j**) Relative free GFP levels in panel **i**. The ratio of free GFP to actin in the control WT was set to 1. For the western blotting assay, actin was employed as a loading control. Data reflect the mean of three biological replicates (± standard deviation). All studies were performed three times and similar findings were obtained each time. Different lower-case letters represent significant differences at *P* < .05 according to Tukey’s test. FW, fresh weight.

To further analyze the biological functions of autophagy under LN conditions, we detected the phenotype and autophagy accumulation in two *ATG6* knockout mutants (*atg6-1* and *atg6-8*) and two overexpression lines (*ATG6*-OE1 and *ATG6*-OE2) (Supplementary Data Fig. S1). As shown in Fig. 2a, the phenotypes did not show significant differences among the mutant, WT, and OE lines under N-control conditions. After 2 weeks of LN treatment, the leaves of *atg6* mutants began to lose their green color, but the WT and *ATG6*-OE plants remained green (data not shown). After 3 weeks, the leaves of *atg6* mutants showed chlorosis, the leaves of WT plants were light green, and the leaves of *ATG6*-OE plants remained green (Fig. 2a). Similar to the observed phenotypes, the biomass and chlorophyll content were not significantly different among plants under N-sufficient conditions (Fig. 2b and c). Moreover, the plant biomass and chlorophyll content in *atg6*, WT, and *ATG6*-OE plants after 3 weeks of LN stress were all lower than those in control plants. The seedling biomass of the *atg6-1* and *atg6-8* mutants was 20.7 and 21.8% lower, and the chlorophyll content in these mutants was 41.1 and 35.7% lower, respectively, than those in the WT plants after LN stress, while the seedling biomass in the *ATG6*-OE1 and *ATG6*-OE2 lines was 21.6 and 18.5% higher, and the chlorophyll content in these OE plants was 13.7 and 11.0% higher, respectively, than those in the WT plants (Fig. 2b and c).

To further study ATG6 function in autophagosome formation under LN stress, we measured the autophagic activities in both the leaves and roots of *atg6*, WT, and *ATG6*-OE seedlings through classical MDC- and GFP-ATG8f-labeling methods. Few MDC-labeled autophagosomes were observed in either the leaves or roots of any of the plants under N-control conditions (Fig. 2d–g). By contrast, the numbers of MDC-stained autophagosomes were increased 29.8- and 35.7-fold in WT leaves and roots, respectively, after 5 days of LN stress (Fig. 2d–g). Under LN stress, the formation of autophagosomes was substantially reduced in *atg6* mutants, while it was induced in *ATG6*-OE plants to a higher degree than in WT plants (Fig. 2d–g). In agreement with the MDC staining results, we observed that the formation of GFP-labeled punctate autophagosomes markedly increased, by 42.8-fold, in WT roots after LN stress (Supplementary Data Fig. S3A and B). The number of GFP-labeled punctate autophagosomes increased by only 1.5- and 1.4-fold in *atg6-1* and *atg6-8* roots, but increased by 105.5- and 146.6-fold in *ATG6*-OE1 and *ATG6*-OE2 roots after 3 weeks of LN stress (Supplementary Data Fig. S3A and B). To further verify the autophagic activities, we performed western blotting to detect the protein levels of ATG8-PE and free GFP. The level of ATG8-PE in both the leaves and roots of WT plants increased after the onset of LN stress (Fig. 2h). Importantly, the knockout of *ATG6* dramatically suppressed, while the overexpression of *ATG6* further increased, the abundance of ATG8-PE compared with that in WT plants under LN conditions (Fig. 2h). LN stress massively boosted GFP-ATG8f degradation, as shown by the accumulation of free GFP (Fig. 2i and j). Importantly, the abundance of free GFP in *ATG6*-OE plants was more pronounced than that in WT and *atg6* mutants under LN conditions (Fig. 2i and j). These results indicate that ATG6-dependent autophagy is essential in the response of tomato to N-limited conditions.

### Autophagy is related to nitrogen absorption under low-nitrogen stress

**Figure 3:**
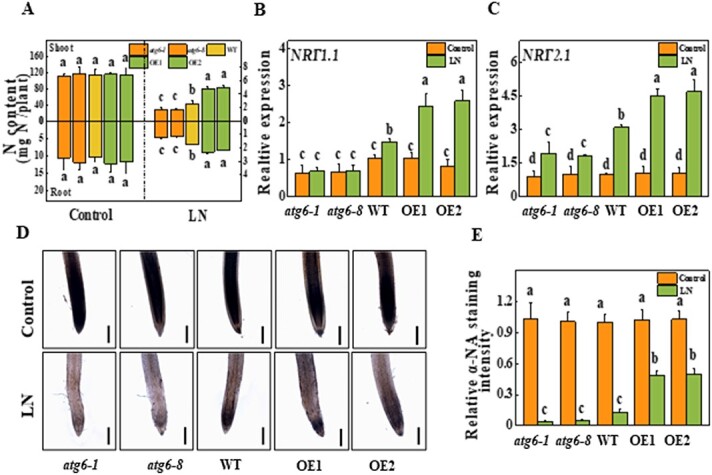
Role of ATG6-dependent autophagy in N uptake under LN stress. **a** N contents of the shoots and roots. Four-week-old plants were transplanted to LN solution for 3 weeks. *NRT1.1* (**b**) and *NRT2.1* (**c**) expression in response to N deficiency on the third day under LN stress. For qRT–PCR analysis, total RNA was extracted from roots. **d** Viability of root tips by α-naphthylamine staining. Samples were examined on the third day under LN stress. Scale bars = 300 μm. **e** Quantitative assay of root viability according to the intensity of α-naphthylamine (α-NA) staining. More than 20 images were measured. Relative integrated optical density per image was quantified to calculate root viability relative to WT control plants, which was set to 1. Data represent the mean of three biological replicates (± standard deviation). All studies were performed three times and similar findings were obtained each time. Different lower-case letters represent significant differences at *P* < .05 according to Tukey’s test. OE1 and OE2, two *ATG6*-overexpressing lines.

To analyze the function of ATG6-dependent autophagy in N absorption and transport, we determined the N content of tomato shoots and roots. The N contents of both shoots and roots were not significantly different among *atg6*, WT, and *ATG6*-OE plants under N-sufficient conditions (Fig. 3a). However, the N contents decreased dramatically, by 97.7% and 83.1%, in the shoots and roots of WT plants after 3 weeks of LN stress compared with those in the WT control plants (Fig. 3a). Strikingly, the N content in the shoots of *atg6-1* and *atg6-8* mutants was 33.1 and 33.0% lower, and that in the roots was 30.3 and 34.1% lower, respectively, than that in WT plants under LN stress (Fig. 3a). Although LN decreased the N content in the *ATG6*-OE plants, the N content in the shoots of *ATG6*-OE1 and *ATG6*-OE1 lines was 33.1 and 33.0% higher, and that in the roots was 30.3 and 34.1% higher, respectively, than that in the WT plants (Fig. 3a).

To further survey the role of ATG6-dependent autophagy in N absorption, we examined the transcript levels of various nitrate transporters in the roots of *atg6*, WT, and *ATG6*-OE plants. The expression of dual-affinity *NRT1.1* and high-affinity *NRT2.1* was not significantly different among all seedlings under N-sufficient conditions (Fig. 3b and c). Compared with those in the control treatment, the transcripts of *NRT1.1* and *NRT2.1* in the roots of WT were 41.6 and 209.9% higher under the LN treatment. Importantly, LN-induced expression of *NRT1.1* and *NRT2.1* was completely compromised in the roots of the *atg6-1* and *atg6-8* mutants. However, the expression of *NRT1.1* and *NRT2.1* was increased by 64.7 and 45.8%, respectively, in the *ATG6*-OE1 line and by 74.4 and 51.9% in the *ATG6*-OE2 line compared with that in WT plants under LN stress (Fig. 3b and c). As root viability is critical for N absorption, we examined the viability of the root cells by determining the *α*-naphthylamine-oxidizing activity (*a-*NOA), which can be used as an indicator of root vigor [28]. Under N-sufficient conditions, no notable differences in root viability were identified among the plants; however, LN stress markedly reduced the vigor of all roots (Fig. 3d and e). Furthermore, the root vigor in *atg6-1* and *atg6-8* decreased by 70.0 and 61.7%, respectively, while that in *ATG6*-OE1 and *ATG6*-OE2 plants increased by 2.7- and 2.8-fold, respectively, compared with that in WT plants under LN stress (Fig. 3d and e). The above results indicate that ATG6-dependent autophagy promotes N absorption and alleviates oxidative damage in tomato roots under LN stress.

### Autophagy modulates nitrogen assimilation and protein accumulation under low-nitrogen stress

**Figure 4:**
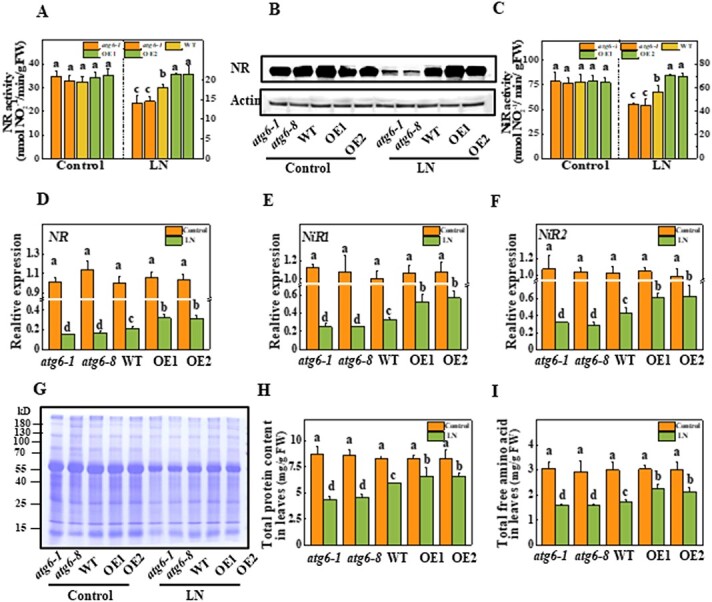
Role of ATG6-dependent autophagy in N assimilation and protein accumulation under LN stress. **a** Activities of NR in the leaves of tomato plants on the third day under LN stress. **b** Accumulation of NR proteins in the leaves on the third day under LN stress. For the western blotting assay, actin was employed as a loading control. **c** Activities of NiR in the leaves of tomato plants on the third day under LN stress. *NR* (**d**), *NiR1* (**e**), and *NiR2* (**f**) expression in response to N deficiency on the third day under LN stress. Total RNA was extracted from leaves and used for qRT–PCR analysis. **g** Total protein levels. Total proteins based on 0.1 g of fresh weight from upper functional leaves were used for SDS–PAGE and stained with Coomassie blue after 5 days of LN stress. **h** Quantitative assay of total proteins in panel **g**. Leaf samples were dyed with Coomassie blue solution and absorption at a wavelength of 595 nm was determined by ultraviolet spectrophotometry. **i** Content of total free amino acids in the leaves. Data reflect the mean of three biological replicates (± standard deviation). All studies were performed three times and similar findings were obtained each time. Different lower-case letters represent significant differences at *P* < .05 according to Tukey’s test. OE1 and OE2, two *ATG6*-overexpressing plants; FW, fresh weight.

To further investigate the role of ATG6-dependent autophagy in N assimilation, we determined the activity and expression of NR and NiR in the leaves. As shown in Fig. 4a–f, the activity and transcript levels of NR and NiR were similar across all plants under N-sufficient conditions. LN treatment inhibited the activity and transcript levels of *NR* and *NiR* in WT plants. Importantly, NR activity in *atg6-1* and *atg6-8* plants decreased by 21.7 and 18.6%, respectively, while that in *ATG6-*OE1 and *ATG6-*OE2 plants increased by 19.2 and 20.3%, respectively, compared with that in WT plants after LN treatment (Fig. 4a). Moreover, the accumulation of NR proteins decreased slightly in WT plants, was induced in *ATG6*-OE plants, and was significantly inhibited in *atg6* mutants after 3 days of LN stress (Fig. 4b). Additionally, NiR activity in *atg6-1* and *atg6-8* plants decreased by 18.3 and 20.1%, respectively, while that in *ATG6-*OE1 and *ATG6-*OE2 increased by 25.6 and 23.7%, respectively, compared with that in WT plants after 3 days of LN stress (Fig. 4c). The expression patterns of *NR*, *NiR1* and *NiR2* were consistent with the results for NR and NiR activity (Fig. 4d–f).

Proteins and amino acids are the main products of N assimilation. We next investigated the contents of proteins and amino acids in plant leaves. Consistent with the total N accumulation, the contents of proteins and amino acids were not significantly different among plants under N-sufficient conditions, but they both decreased under LN conditions in all plants (Fig. 4g–i). Importantly, under LN stress, the contents of proteins and amino acids increased in the *ATG6*-OE lines but decreased in the *atg6* mutants compared with those in the WT plants (Fig. 4g–i). Further analysis of specific amino acids revealed that under LN stress the amounts of isoleucine, leucine, tyrosine, phenylalanine, lysine, serine, glutamic acid, and glutamine increased in the *ATG6*-OE lines but decreased in the *atg6* mutants compared with those in the WT plants (Supplementary Data Fig. S4). However, the differences in the contents of other abundant amino acids, such as aspartic acid, asparagine, and alanine, were not significant among *atg6*, WT, and *ATG6*-OE plants under LN stress (Supplementary Data Fig. S4). Together, these results indicate that autophagy is involved in N transport and assimilation under LN conditions.

### Autophagy regulates photosynthetic CO_2_ assimilation in response to low-nitrogen stress

**Figure 5:**
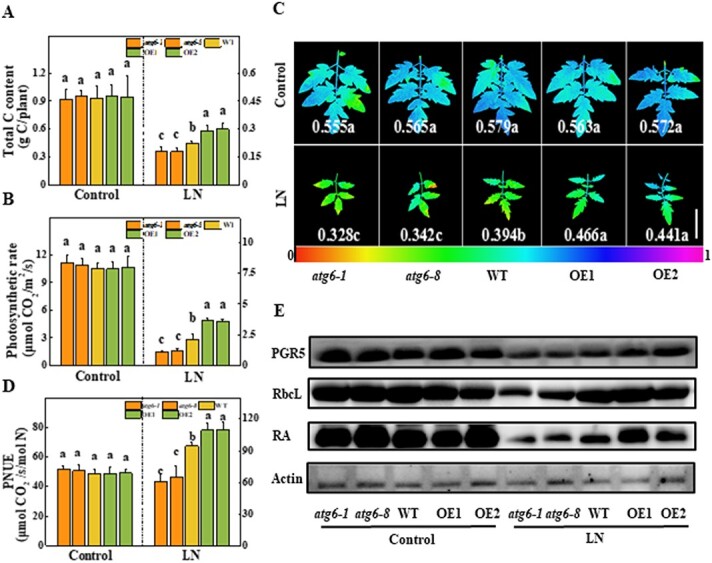
Role of ATG6-dependent autophagy in C fixation and photosynthesis under LN stress. **a** C content of the total plant. Four-week-old plants were transplanted to LN solution for 3 weeks. **b** Leaf photosynthetic rate on the 14th day under LN stress. **c** Changes in photosynthetic efficiency were assessed by determining Φ_PSII_. Scale bar = 5 cm. **d** PNUE was calculated based on the photosynthesis rate relative to the amount of N in leaves of the same size. **e** Immunological detection of RbcL, RA, and PGR5 in tomato leaves. Protein extracts were obtained on the fifth day under LN stress. Data reflect the mean of three biological replicates (± standard deviation). All studies were performed three times and similar findings were obtained each time. Different lower-case letters represent significant differences at *P* < .05 according to Tukey’s test. OE1 and OE2, two *ATG6*-overexpressing plants.

N deficiency leads to a general inhibition of CO_2_ assimilation and plant growth. Thus, we investigated the accumulation of C in *atg6*, WT, and *ATG6-*OE plants. Overexpression of the *ATG6* gene dramatically increased the C content under LN stress, while knockout of the *ATG6* gene decreased it compared with that in the WT plants (Fig. 5a). Under LN conditions, the total C content in *ATG6-*OE1 and *ATG6-*OE2 plants was 28.9 and 32.6% higher, respectively, than that in WT plants, while that in *atg6-1* and *atg6-8* plants was 20.5 and 20.6% lower, respectively (Fig. 5a). Consistent with the C content, the plant photosynthetic parameters, including the photosynthetic CO_2_ assimilation rates, quantum yield of PSII (Φ_PSII_) and maximal photochemical efficiency of PSII (*F*v/*F*m), were inhibited under LN conditions (Fig. 5b and c; Supplementary Data Fig. S5). Strikingly, under LN stress, these photosynthetic parameters were improved in *ATG6*-OE plants but were noticeably compromised in *atg6* mutants compared with those in WT plants (Fig. 5b and c; Supplementary Data Fig. S5). The *F*v/*F*m value in *atg6-1* and *atg6-8* plants decreased by 16.8 and 13.2%, respectively, while that in ATG6-OE1 and ATG6-OE2 plants increased by 18.3 and 12.0%, respectively, compared with that in WT plants under LN treatment (Fig. 5c). The photosynthetic NUE (PNUE) is linked to the ratio of leaf N consumed for C fixation per unit of leaf area and can be used to characterize the amount of N needed for carbohydrates, energy, and structural component formation, as well as plant development [29]. Under LN conditions, the PNUE was 15.6 and 15.6% higher in the *ATG6-*OE1 and *ATG6-*OE2 plants than in the WT plants, while it was 35.7 and 31.2% lower in the *atg6-1* and *atg6-8* mutants (Fig. 5d); these results suggest that ATG6-dependent autophagy promotes NUE under LN conditions.

To further investigate the function of ATG6 in plant photosynthesis under LN stress, we analyzed the accumulation of key photosynthesis proteins in different genotypes under LN stress. The accumulation of proton gradient regulation 5 (PGR5), which regulates PSI cyclic electron flow, and of Rubisco large subunit (RbcL) and Rubisco activase (RA), which catalyze the carboxylation of the 5-C sugar ribulose-1,5-bisphosphate (RuBP) to fix atmospheric CO_2_, was not significantly different among *atg6*, WT, and *ATG6*-OE plants under N-sufficient conditions. N deficiency significantly inhibited the accumulation of these proteins in WT plants (Fig. 5e). Interestingly, under LN stress, the accumulation of PGR5, RbcL, and RA increased in the *ATG6*-OE plants compared with that in the WT plants, but greatly decreased in *atg6* mutants (Fig. 5e). These results indicated that ATG6-dependent autophagy promotes the assimilation of both N and C and subsequently contributes to plant growth under LN stress.

**Figure 6:**
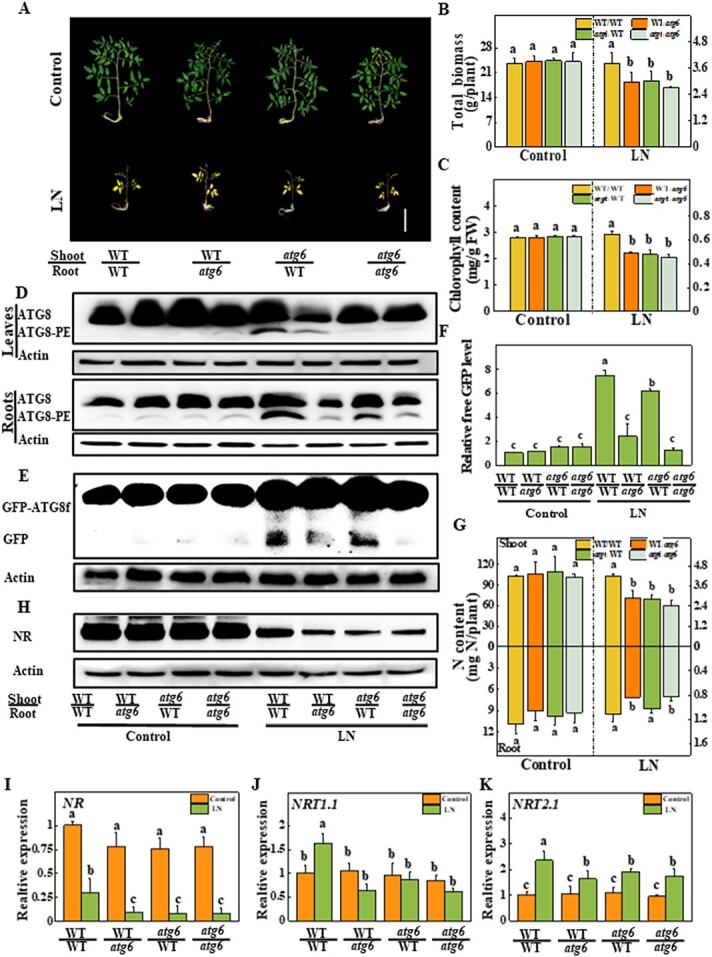
N uptake and utilization are inhibited in *atg6*-grafted plants under LN stress. **a** Phenotypes of *atg6-* and WT-grafted plants. Six-week-old plants were transplanted to LN solution for 3 weeks. Total biomass of the whole plant (**b**) and chlorophyll content of the fourth expanded leaf (**c**) were determined on the 21st day under LN stress. FW, fresh weight. **d** ATG8 lipidation level in leaves and roots on the fifth day under LN stress. ATG8 (non-lipidated form) and ATG8-PE (lipidated form) are indicated on the left. **e** Accumulation of GFP-ATG8f proteins in *GFP-ATG8f-*overexpressing roots of grafted plants on the fifth day under LN stress. GFP-ATG8f fusion and free GFP positions are marked on the left. **f** Relative free GFP levels in panel **e**. The ratio of free GFP to actin in the control WT was set to 1. For the western blotting assay, actin was employed as a loading control. **g** N contents of shoots and roots. Six-week-old plants were transferred to LN medium for 3 weeks. **h** Accumulation of NR proteins in leaves on the third day under LN stress. For the western blotting assay, actin was employed as a loading control. Expression of *NR* (**i**), *NRT1.1* (**j**), and *NRT2.1* (**k**) in response to LN stress on the third day. Data represent the mean of three biological replicates (± standard deviation). All studies were performed three times and similar findings were obtained each time. Different lower-case letters represent significant differences at *P* < .05 according to Tukey’s test.

### Autophagy-mediated nitrogen uptake in roots and utilization in shoots are required for plant growth and photosynthetic CO_2_ assimilation

To further detect the functions of ATG6-dependent autophagy in N uptake, utilization and plant growth under LN conditions, *atg6-1* (hereafter abbreviated as *atg6*) mutants were reciprocally grafted with WT plants as scions or rootstocks. As shown in Fig. 6a, no significant differences in phenotype were observed among the grafted seedlings under N-sufficient conditions. However, the leaves of WT shoots grafted onto *atg6* roots (WT/*atg6*), *atg6* shoots grafted onto WT roots (*atg6*/WT), and *atg6* self-grafted plants (*atg6*/*atg6*) showed chlorosis, while the leaves of WT self-grafted plants (WT/WT) were light green after 3 weeks of LN conditions (Fig. 6a). The total biomass of WT/*atg6*, *atg6*/WT, and *atg6*/*atg6* plants was 22.1, 20.1, and 28.8% lower than that of WT/WT plants under LN stress, respectively (Fig. 6b). Moreover, the chlorophyll content was 24.6, 26.4, and 29.7% lower in WT/*atg6*, *atg6*/WT, and *atg6*/*atg6* plants than in WT/WT plants, respectively (Fig. 6c). Root N accumulation was inhibited when *atg6* mutants were used as rootstocks, while shoot N accumulation was inhibited when *atg6* mutants were used as either scions or rootstocks (Fig. 6g). Importantly, LN-induced autophagic activities were compromised in the roots of plants with *atg6* mutants as rootstocks, while they were inhibited in the leaves of plants with *atg6* mutants as either rootstocks or scions; autophagic activities were measured by detecting the levels of ATG8-PE, free GFP, MDC-stained autophagosomes, and GFP-ATG8f-labeled autophagosomes (Fig. 6d–f; Supplementary Data Fig. S6). Crucially, the accumulation of NR protein and the expression of *NR*, *NiR1*, and *NiR2* were further inhibited in the leaves of WT/*atg6*, *atg6*/WT, and *atg6*/*atg6* plants compared with those in the leaves of WT/WT plants after LN stress (Fig. 6h and i; Supplementary Data Fig. S7). Moreover, the transcription levels of *NRT1.1* and *NRT2.1* in the roots were promoted by LN stress in the roots of WT/WT plants but were fully or partly inhibited in the roots of WT/*atg6*, *atg6*/WT, and *atg6*/*atg6* plants (Fig. 6j and k). Therefore, ATG6-dependent autophagy is required for N uptake in the roots as well as for the subsequent N assimilation in the shoots.

**Figure 7:**
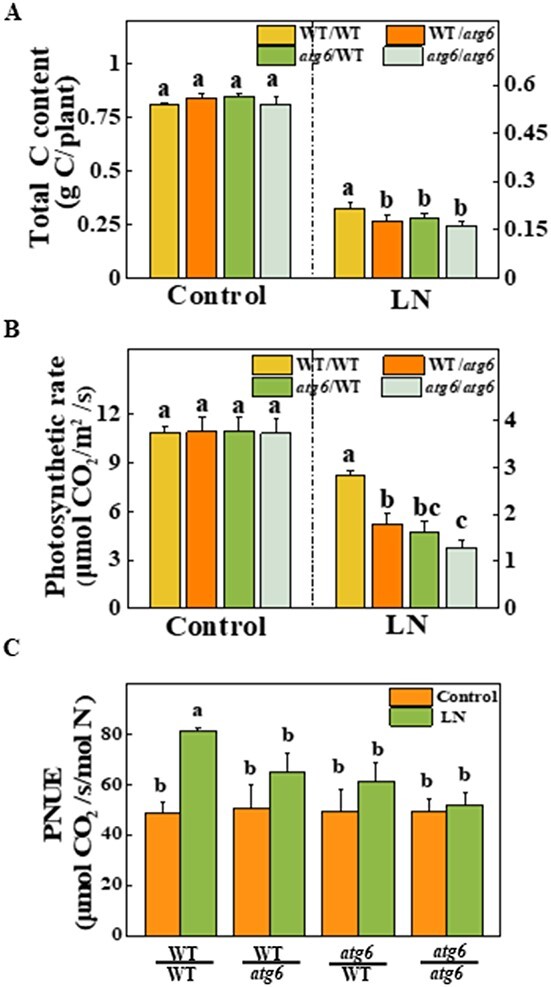
Carbon accumulation and PNUE in *atg6*-grafted plants is influenced by LN stress. **a** Total C content of *atg6-* and WT-grafted plants. Six-week-old plants were transplanted to LN solution for 3 weeks. **b** Leaf photosynthetic rate on the 14th day under LN stress. **c** PNUE was measured based on the photosynthetic rate relative to the amount of N in leaves of the same size. Data reflect the mean of three biological replicates (± standard deviation). All studies were performed three times and similar findings were obtained each time. Different lower-case letters represent significant differences at *P* < .05 according to Tukey’s test.

To further confirm the role of ATG6-dependent autophagy in response to LN conditions, we next examined the total C content in the grafted plants. As shown in Fig. 7a, the C content was significantly lower, by 18.7, 14.4, and 24.2%, in WT/*atg6*, *atg6*/WT, and *atg6*/*atg6* plants, respectively, than in WT/WT plants under LN stress (Fig. 7a). Furthermore, compared with those of WT/WT plants, the photosynthetic rates of WT/*atg6*, *atg6*/WT, and *atg6*/*atg6* plants were 37.2, 42.6, and 54.6% lower, respectively (Fig. 7b). The grafted plants that used *atg6* mutants as either rootstocks or scions all exhibited lower PNUE than WT/WT plants under LN stress (Fig. 7c). Taken together, these findings suggest that ATG6-dependent autophagy systematically improves N metabolism, photosynthesis, and plant growth under LN conditions.

## Discussion

### Autophagy regulates nitrogen uptake under low-nitrogen conditions

Previous studies have shown that numerous *atg* mutants display strong, early leaf senescence symptoms and are sensitive to LN stress [19, 20, 22, 30]. The functions of autophagy in N remobilization were impaired in *atg* mutants, e.g. maize *atg12* and *Arabidopsis atg5* and *atg9*, leading to lower N accumulation and yields in the storage organs of *atg* mutants [19, 20]; in contrast, the overexpression of *ATG18a* enhanced leaf nitrate content and LN stress tolerance in apple [21]. In this study, the expression of *ATG* genes was elevated in tomato plants under LN conditions; moreover, multiple tomato *atg* mutants displayed hypersensitivity to N limitation, exhibiting biomass reduction followed by accelerated leaf chlorosis (Fig. 1).

ATG6 is the major component of the PI3K complex that is located at phagophore assembly sites; it is an essential component in the formation of phagophores by engaging other effector proteins [31]. For instance, *ATG6*-silenced tomato plants showed severe inhibition of the brassinosteroid-induced formation of autophagosomes [22]. In addition, ATG6 also interacts with other proteins to regulate autophagic processes in plants. For instance, Bax inhibitor-1 (BI-1) in tobacco interacted with ATG6 to trigger autophagy and programmed cell death, while *ATG6* silencing inhibited BI-1-induced autophagic activity and autophagy-dependent cell death [32]. In the present study, we found that ATG6 mediated autophagy activity in both roots and leaves under LN stress (Fig. 2). Moreover, ATG6-dependent autophagy improved root viability and N uptake by increasing the expression of *NRT1.1* and *NRT1.2* under LN stress (Fig. 3). Similarly, the overexpression of apple *ATG18a* also upregulated three high-affinity nitrate transporters, NRT2.1/2.4/2.5, in response to LN conditions [21]. Therefore, when N starvation occurs, a high level of autophagy not only benefits N recycling in plants but also positively regulates N uptake. We propose a hypothetical molecular mechanism by which selective autophagy can directly/indirectly target the upstream factors of *NRTs* and thus regulate N uptake. However, direct molecular evidence still needs to be elucidated.

Early signals, such as reactive oxygen species (ROS), nitric oxide (NO), and calcium ions (Ca^2+^), are critical for both autophagy induction and N absorption under stress conditions [33–35]. ROS targeted the autophagy upstream regulator SNF1-related protein kinase 1 (SnRK1) to activate autophagy [36]. Furthermore, ROS reversibly inhibited ATG4 proteases to ensure the lipidation of ATG8 during autophagosome biogenesis in *Arabidopsis* under stress conditions [37]. Moreover, ROS scavenging inhibited the induction of *NRT2.4* and *NRT2.5* in *Arabidopsis* under N starvation [38]. NO signaling is linked to autophagy via its main regulator, *S*-nitrosoglutathione reductase 1 (GSNOR1). GSNOR1 is *S*-nitrosylated and then interacts with ATG8 to be degraded by selective autophagy during hypoxia responses in *Arabidopsis* [39]. GSNOR can also upregulate the transcription levels of *NRT2.1* and *NR* [40]. The Ca^2+^ signaling protein calmodulin-like 24 (CML24) can interact with ATG4b; *cml24* mutants showed aberrant ATG4 activity patterns and altered ATG8 accumulation levels, leading to increased sensitivity to prolonged darkness [41]. The calcium sensor factor calcineurin B-like protein (CBL)-interacting protein kinase 23 (CIPK23) can phosphorylate NRT1.1 to activate the high-affinity mode of NRT1.1 under low-nitrate conditions, which allows plants to adapt to such conditions [42]. Considering the close association between second messengers and autophagy in the N absorption process, it is tempting to speculate that autophagy-mediated N uptake may be related to early signals, such as ROS, NO, or calcium levels.

### Autophagy improves nitrogen transport and assimilation under low-nitrogen conditions

Previous studies have shown that *atg* mutants inhibit N redistribution in plants and alter the composition of N metabolites [30]. However, the results of such studies have focused on the roles of autophagy in nutrient recycling, and it remains unknown whether or how autophagy improves N transport and assimilation. In addition to N absorption enhancement, we determined that ATG6-dependent autophagy is involved in N assimilation via its regulation of the key nitrate assimilation enzymes NR and NiR. Moreover, ATG6-dependent autophagy increased the contents of proteins and amino acids under LN stress (Fig. 4). Intriguingly, SnRK1, an upstream kinase for autophagy, can initiate autophagy by directly phosphorylating ATG1 or by phosphorylating ATG6 under C starvation [43]. Moreover, SnRK1 regulates N metabolism [44]. Knockout of a subunit of SnRK1 complexes, *AKINβ1*, inhibited the expression of *NTR1.8*, which primarily influences long-distance nitrate transport from roots to shoots under both light and darkness conditions in *Arabidopsis* [45]. AKINβ1 has also been shown to interact with and phosphorylate NR to negatively regulate its activity [46, 47]. Therefore, autophagy-mediated N transport and assimilation may be activated by SnRK1 and crosstalk with SnRK1-regulated N metabolism.

The transport of N from roots to shoots involves a variety of complex signaling networks. Through grafting experiments, we found that ATG6-dependent autophagy in the roots was essential for N transport from roots to shoots, and *atg6* mutants used as rootstocks showed reduced N accumulation and PNUE in combination with WT scion shoots (Figs 6 and 7). Intriguingly, *atg6* mutants used as rootstocks also interfered with the formation of autophagosomes in WT scions under LN stress (Fig. 6). These findings indicate that autophagy plays an essential role in N transport and assimilation. Previous studies have shown that some systemic signaling molecules are involved in N transport and utilization [48]. The cytokinin transzeatin (tZ) is a component of the systemic signaling pathway derived from nitrate; tZ is induced by the nitrate supply in *Arabidopsis* roots and translocated to shoots to regulate gene expression, including that of genes involved in glutamate and glutamine biosynthesis [49]. Cytokinin mutants had lower root biomass and nitrate absorption, as well as decreased systemic nitrate signaling responses [49]. The peptide transport system also acts as a root-to-shoot signal to regulate *NRT* expression under N starvation conditions. Under N-poor conditions, C-terminally encoded peptides (CEPs) were biosynthesized and upregulated in the roots and translocated to shoots to activate the production of a second class of peptides, CEP downstream (CEPD), which were subsequently transmitted to roots to induce *NRT2.1* expression and nitrate uptake [50]. Interestingly, systemic signals are also associated with autophagy. Selective autophagy mediated by the exocyst subunit Exo70 family protein (EXO70D) can target and degrade the negative regulators of cytokinin signaling type-A response regulators (type-A ARRs) in *Arabidopsis* roots [51], indicating the modulation of cytokinin signaling by selective autophagy. Therefore, autophagy may act as a positive regulator of the plant signal sensing system in response to N deficiency stress.

### Significance of autophagy for carbon fixation under low-nitrogen conditions

The photosynthetic capacity of leaves has a strong positive correlation with their N content, and most N is used for the synthesis of photosynthetic components [52, 53]. Under LN stress, less N was allocated to chlorophyll and light-harvesting proteins in maize leaves [53, 54]. In addition, N deficiency decreased the photochemical efficiency of PSII as well as the electron transport rate (ETR) in rice [55]. Interestingly, Barros *et al.* [56] suggested that autophagy deficiency accelerated the loss of photochemical efficiency in a markedly early-senescence phenotype in *Arabidopsis* during extended darkness. Our data support this finding, as we observed lower values of the key PSII parameters Φ_PSII_ and *F*v/*F*m in *atg6* mutants under LN stress, while ATG6-dependent autophagy alleviated LN-induced photosynthetic damage (Fig. 5; Supplementary Data Fig. S5). Thus, autophagy may be involved in PSII photoprotection and repair under LN stress.

Autophagy is also involved in the regulation of photosynthetic enzymes as part of the plant response to nutrient deficiency. The main photosynthetic enzyme, Rubisco, can be mobilized to the vacuole to be degraded under starvation conditions through an *ATG*-dependent autophagic process without prior chloroplast destruction in *Arabidopsis* [57]. Moreover, autophagy increases photosynthesis by degrading the damaged photosystem apparatus under stress. *Arabidopsis atg* mutants exhibited UV-B-sensitive phenotypes and accumulated collapsed chloroplasts through oxidative damage, indicating that the autophagy-mediated removal of damaged chloroplasts facilitates photosynthesis and stress tolerance [58]. The abundance of proteins associated with photosynthesis and the size of chloroplasts were reduced in maize *atg12* mutants, leading to premature senescence of plants under N-limited conditions [59]. Therefore, autophagy regulates photosynthesis in part by recycling photosynthetic enzymes and chloroplast components under nutrient stress. Nevertheless, our experiments suggest that autophagy has more functions than merely the degradation and recycling of organelles and proteins. The accumulation of photosynthetic enzymes RbcL and RA and PGR5 proteins was increased by ATG6-dependent autophagy under LN stress. ATG6-dependent autophagy also promoted photosynthesis and PNUE by optimizing N management under N-limited conditions. Moreover, grafting of a WT scion onto *atg6* rootstock and an *atg6* scion onto WT rootstock resulted in compromised C fixation and PNUE in tomato seedlings under LN stress (Fig. 7). This suggests that autophagy mediates the systematic coregulation of photosynthesis and N utilization under LN stress.

In conclusion, our results reveal new functions of autophagy, i.e. regulating N uptake and utilization as well as C assimilation, in addition to nutrient recycling and remobilization in tomato under LN stress. Thus, intentionally enhancing autophagy may be a beneficial strategy for improving crop growth and yield under nitrogen-deficient conditions. In addition, future research should be undertaken to reveal the precise regulatory mechanisms of autophagy with regard to N uptake and assimilation.

## Materials and methods

### Plant materials and growth conditions

In this work, the ‘Ailsa Craig’ (AC) tomato (*Solanum lycopersicum*) and transgenic plants generated on its background were used. Experiments were carried out on two independent homozygous *ATG6*-overexpressing lines, which were identified using western blot with an anti-hemagglutinin (anti-HA; Thermo Fisher Scientific, 26183) monoclonal antibody. To create the *ATG6-*overexpresing construct, the *ATG6* coding sequence (CDS) was amplified using AC tomato cDNA as the template with the primers (5′-ttggcgcgccATGGTGAAAGGCAGCAGCG-3′) and (5′-ggggtaccAGATTGAAACTTGGTATT-3′). The full-length CDS was inserted into the pFGC1008-HA vector with the CaMV 35S promoter. The study also used two independent *atg6* lines: one *atg10* line and one *atg18a* line that included mutations in exons and generated premature stop codons resulting in truncated proteins. The *atg6*, *atg10*, and *atg18a* CRISPR/Cas9 vectors were constructed as described previously [60]. The target sequences (*ATG6* 5′-GGTAAAGTCCGACCCTTATCCGG-3′; *ATG10* 5′-AAGTTGATCCATGACCAGTGAGG-3′; *ATG18a* 5′-TGGGTGCTGAACTGGTGGGGGG-3′), obtained by using the CRISPR-P network tool (http://crispr.hzau.edu.cn/), were annealed to double-stranded DNA and inserted into the BbsI site of the AtU6-sgRNA-AtUBQ-Cas9 vectors. These fragments were introduced into pCAMBIA1301 binary vectors. All of the above plasmids were electropermeabilized into *Agrobacterium tumefaciens* strain EHA105 and inoculated onto AC cotyledons. Independent homozygous T2 gene editing mutations were selected in subsequent experiments and Cas9 was isolated. For the generation of tomatoes with transgenic roots, the full-length CDSs of *GFP* and tomato *ATG8f* were used as described previously [27]. To obtain the *ATG8f-*overexpressing construct, *GFP* and the *ATG8f* full-length cDNA sequence were amplified with the primers (*GFP* 5′-TTggcgcgccATGGTGAGCAAGGGCGAG-3′, 5′-GAATGAGCTCTTAGCCATCTTGTACAGCTCGTCC-3′ and *ATG8f* 5′-GGACGAGCTGTACAAGATGGCTAAGAGCTCATTCAAG-3′, 5′-CGCggatccCTACAGTTCGCTCAGGACC-3′) by overlapping PCR and introduced into the pFGC1008 vector. This plasmid was electropermeabilized into *Agrobacterium rhizogenes* strain K599 (Tolobio, CC96315) for inoculation of the hypocotyls of the aseptic tomato seedlings, which were WT, *atg6*/*atg10*/*atg18a* mutants, and *ATG6*-overexpressing lines. The specific steps of genetically modified tomato hair roots were as previously described [27]. Briefly, tomato seeds were sown in 1/2 Murashige and Skoog medium (PhytoTechnology, M519), and after 5–7 days of seedling growth the tap roots were removed and their hypocotyls were infected with *A. rhizogenes* strain K599. After coculture with K599, they regenerated root systems. The primary roots were excised again and the hypocotyls were expanded to obtain hairy roots that overexpressed GFP-*ATG8f* and were transplanted into the nutrient solution for subsequent experiments.

After germination, seeds were sown in 250-cm^3^ plastic pots loaded with a perlite–vermiculite mixture (2:1, v:v). The plants were grown in a growth chamber and were hydrated daily with Hoagland’s nutrition solution. The growth parameters were 25/20°C day/night temperature and a photoperiod of 12 h with 400 μmol m^−2^ s^−1^ photosynthetic photon flux density (PPFD). For N-limiting experiments, 3-week-old seedlings were cultured in the control/N-limited solution, which was refreshed every other day. Nutrient solutions contained two different levels of nitrate: (i) control solutions, according to Hoagland’s nutrient formula with slight modification, containing 10 mM NO_3_^−^ [61]; and (ii) LN solutions only containing 0.1 mM NO_3_^−^, other components correspondingly being 3.75 mM CaCl_2_, 1.5 mM KH_2_PO_4_, 2.7 mM K_2_SO_4_ and 1 mM MgSO_4_·7H_2_O; the pH of both solutions was adjusted to 6.0 with NaOH. In all solutions, the trace elements were provided as described previously [62].

For grafting experiments, two-leaf-stage seedlings of WT and *atg6-1* (hereafter abbreviated as *atg6*) were self-grafted and intergrafted, respectively, resulting in four lines of grafted plants: WT/WT, WT/*atg6*, *atg6*/WT, and *atg6*/*atg6*. After 3 days of adaptation in the dark, grafted seedlings were progressively exposed to light (reaching a 400 μmol m^−2^ s^−1^ PPFD with 25/20°C temperatures). Then, well-growing plants were chosen for the subsequent experiments.

### Total RNA extraction and gene expression analysis

RNA extraction kits (Tiangen, DP419) were used to extract total RNA from plant leaves and roots. HiScript Q RT SuperMix for qPCR (+gDNA wiper) Kits (Vazyme, R223) were used to create first-strand cDNA from 500 ng of total RNA. We performed qRT–PCR with a Light Cycler^®^ 480 II Real-Time PCR detection system (Roche, Germany). The PCR procedure was according to the instructions for the kit. Supplementary Data Table S1 lists the primers used for qRT–PCR, and internal controls used the tomato genes *Actin* and *Ubiquitin 3*. Relative gene expression was computed by the 2^-△△Ct^ method as described previously [63]. The heat map analysis was conducted using MeV version 4.9. The degree of gene expression was indicated by color bar intensity at the bottom of the MeV viewer.

### Measurement of autophagy

MDC staining of tomato leaf and root samples was conducted as described previously [64]. Briefly, leaf and root samples were dissected and then subsequently vacuum-infiltrated three times with 0.01 mM MDC (Sigma–Aldrich, 30432) and placed in the dark for 30 minutes. After washing twice with phosphate-buffered saline (PBS; Solarbio, P1020), leaves and roots cells were observed using a Nikon A1+ confocal microscope (Nikon, Japan). The excitation wavelength of 405 nm and emission wavelength ranging from 470 to 520 nm were selected for detecting MDC dyeing. Tomato roots overexpressing GFP-*ATG8f* were cut into small sections, and autophagosomes were visualized with a Nikon A1+ confocal microscope (Nikon, Japan). The excitation wavelength of 488 nm and emission wavelength ranging from 493 to 558 nm were selected for detecting GFP-*ATG8f*. For each treatment, 10–30 representative photographs were taken, and the number of fluorescent puncta or autophagic bodies in each image was counted and averaged manually.

### Protein extraction and western blotting assay

Plant protein extraction was assayed as described previously [22]. Tomato leaf and root samples were crushed and extracted using protein extraction buffer [50 mM Tris–HCl (pH 7.5), 1 mM EDTA, 150 mM NaCl, 1 mM PMSF, 10 mM DTT, 0.2% Triton-100]. All samples were denatured with 5 × sample loading buffer (FUDE Biological Technology Co., Ltd, FD002) and were separated on 10–12% SDS–PAGE gel. Blots were blocked immediately with agitation for 1 hour in 5% non-fat milk. Washing with 1 × TBST buffer (5 minutes × 5 times) was followed by overnight incubation at 4°C in 1 × TBST buffer with 1% BSA containing first antibody with agitation. The blots were then washed with 1 × TBST buffer (5 min × 5 times) and incubated with the secondary antibody with agitation for 1 h. Blots were washed as above and incubated with ECL reagent (FUDE Biological Technology Co., Ltd, FD8030). The chemiluminescence was recorded with a Bio-Rad Touch Imaging system (Bio-Rad, USA). For lipidated ATG8 protein detection, the denatured plant proteins were separated and subjected to 13.5% SDS–PAGE with 6 M urea [20]. The following first antibodies were used: anti-HA monoclonal antibody (Thermo Fisher Scientific, 26183), anti-actin polyclonal antibody (Abcam, ab197345), anti-GFP polyclonal antibody (Genescript, A01704), anti-ATG8 polyclonal antibody (Agrisera, AS142769), anti-NR polyclonal antibody (Agrisera, AS08310), anti-RbcL polyclonal antibody (Agrisera, AS03037), anti-RA polyclonal antibody (Agrisera, AS10700), and anti-PGR5 polyclonal antibody (Agrisera, AS163985). The following second antibodies were used: goat anti-mouse HRP-linked antibody (Bio-Rad, 170-6516) and goat anti-rabbit HRP-linked antibody (Cell Signaling Technology, 7074). The relative free GFP levels were measured using ImageJ (https://imagej.en.softonic.com/).

### Measurement of total carbon and nitrogen contents

Plant shoots and roots were baked at >100°C for 35 minutes in an oven, and then dried to a constant weight at 65°C for >5 days. Dehydrated plant tissues were milled and weighed, then high-temperature combustion processes were used to determine N and C concentrations [N% and C%, milligrams of N or C per 100 mg dry weight (DW)] by using a Flash IRMS Elemental Analyzer (Thermo Fisher Scientific, USA) as previously described [65]. Total N or C (N/C) content was calculated as DW_shoot_ × N/C%_shoot_ + DW_root_ × N/C%_root_.

### Measurement of root viability

To investigate root viability, *a-*NOA has been used as staining dye [28, 66]. Tomato root tips (1 cm) were infiltrated with 0.25% (w/v) *a-*NOA for 30 minutes, and then the tissues were rinsed with 1 × PBS buffer. The stained root tips were observed using a microscope (Zeiss Axio Scope A1, Germany). The intensity of the staining of roots was quantified with Image-Pro Plus 6.0 (Media Cybernetics, USA).

### Measurement of nitrate reductase and nitrite reductase activity

The activities of NR and NiR were measured according to previous studies [67, 68]. Briefly, frozen leaf samples were ground and exacted with 1.5 ml of extraction buffer [25 mM Tris–HCl (pH 7.5), 1 mM Na_2_MoO_4_, 5 mM DTT, 2 mM β-mercaptoethanol, 5 mM EDTA, and 1% PVPP]. The supernatant was collected for the enzyme activity test after centrifugation. For the NR activity test, the reaction mixture consisted of 0.7 ml buffer [100 mM PBS buffer (pH 7.5), 5 mM KNO_3_, and 0.25 mM NADH] and 0.3 ml enzyme extract. The reaction was incubated for 30 minutes at 25°C and stopped with an equal volume of 1% sulfanilamide and 0.02% *N*-(1-naphthyl)-ethylenediamine dihydrochloride, and the absorbance was recorded by a spectrophotometer at 540 nm. Activity of NR was expressed as the amount of NO_2_^−^ formed. For the NiR activity test, the reaction mixture included 1.2 ml buffer [100 mM PBS buffer (pH 7.5), 0.5 mM NaNO_2_, 2.5 mM methyl viologen, and 15 μM sodium dithionite] and 0.1 ml enzyme extract. The reaction was incubated for 30 minutes at 25°C, and then the 0.1 ml reaction mixture was rapidly fixed volume to 1 ml with distilled water, and the amount of nitrite was determined by adding 0.25 ml 1% sulfanilamide and 0.25 ml 0.02% *N*-(1-naphthyl)-ethylenediamine dihydrochloride. Activity of NiR was expressed as the amount of NO_2_^−^ utilized. The absorbance was measured by spectrophotometer at 540 nm. The amount of NO_2_^−^ was calculated according to a nitrite standard curve.

### Measurement of amino acids

Leaf samples were ground in liquid N_2_ and mixed thoroughly for 2 h at 4°C in 4% sulfosalicylic acid buffer [69]. The supernatant was collected and mixed with 0.02 M HCl in a ratio of 1:4 after centrifugation, and the resulting solution was passed through a disposable sterile filter to remove impurities. The amino acid content was analyzed with an L-8900 High Speed Amino Acid Analyzer (Hitachi, Japan).

### Measurements of chlorophyll content and photosynthetic parameters

Chlorophyll content was analyzed as previously [22]. Chlorophyll was extracted in 80% (v/v) acetone and its content was measured by spectrophotometry. Photosynthetic rates were determined using an Li-6400 Portable Photosynthesis Analysis System (Li-Cor, USA) at 9–11 a.m. with fixed conditions of 800 μmol photons m^−2^ s^−1^ and 450 μl l^−1^ CO_2_. Photosynthetic parameters were determined after 2 weeks of LN treatment. We determined Φ_PSII_ (quantum yield of PSII) and *F*v/*F*m (maximal photochemical efficiency of PSII) using an Imaging-PAM Chlorophyll Fluorometer (Heinz Walz IMAG-MAXI, Germany) as described previously [70].

### Statistical analysis

In each experiment at least three independent replicates were used. The data were expressed as the mean ± standard deviation of independent biological replicates. All the data were statistically analyzed using analysis of variance (ANOVA), and the significance of treatment differences was determined using Turkey’s test at *P* < .05.

## Supplementary Material

Web_Material_uhac068

## Data Availability

The data used to support the findings of this study are available from the corresponding author upon request.
