# Supplementary material for: Autophagic pathway contributes to low-nitrogen tolerance by optimizing nitrogen uptake and utilization in tomato
Source: Hortic Res. 2022 Mar 23;9:uhac068. doi: 10.1093/hr/uhac068 (PMC9164271; doi:10.1093/hr/uhac068)
Supplement: Web_Material_uhac068 [file web_material_uhac068.zip › 20211109 Table S1. Primers used for qPCR assays.docx]

| **Table S1.  Primers used for qPCR assays.** | | | |
| --- | --- | --- | --- |
| Gene name | Accession numbers | Forward primer | Reverse primer |
| *ATG1a* | Sl09g011320 | 5′-AGTTCGGAAAGTCCCTCATC-3′ | 5′-ATGATAGCAGAGGCAGAACG-3′ |
| *ATG2* | Sl01g108160 | 5′-GTCATTGAAGAGGCACTGCT-3′ | 5′-AGCTAAATCAACACGGCAAG-3′ |
| *ATG3* | Sl06g034160 | 5′-GAGAGGAGTTGAACCCGAAG-3′ | 5′-CGAAGGAAGTTGACAGCAAA-3′ |
| *ATG4* | Sl01g006230 | 5′-AATTGATCCCTCCTTGGCTA-3′ | 5′-GATGTGGCAGAGCTACGAGT-3′ |
| *ATG5* | Sl02g036380 | 5′-TCAGATGGTGCTGAGATCAAG-3′ | 5′-ATTGTTTACCACCCATGCAA -3′ |
| *ATG6* | Sl05g050390 | 5′-CCCATGCAGTCAAACAATTC-3′ | 5′-CCCTCATGCATTCAAGACAC-3′ |
| *ATG7* | Sl11g068930 | 5′-ATTCAACGGCTAACCGTACC-3′ | 5′-CAAACTCAGCTTTGGCACAT-3′ |
| *ATG8a* | Sl07g064680 | 5′-ACCGGTGATTGTTGAGAAGG-3′ | 5′-GCGCTGAGCTTAATCCTCTT-3′ |
| *ATG8b* | Sl02g080590 | 5′-GGAGAGGAGGCAGTCAGAAT-3′ | 5′-AGTCAAATCAGCTGGGACAA-3′ |
| *ATG8c* | Sl03g031650 | 5′-TTGGCCAATTTGTTTACGTT-3′ | 5′-AAAGGAATCCGTCTTCATCC-3′ |
| *ATG8d* | Sl10g006270 | 5′-AATATCCTTCCTCCCACAGC-3′ | 5′-GCAGAGAGGTTTGACTGCAT-3′ |
| *ATG9* | Sl04g008630 | 5′-ATGTGCATCCTGAAATCGAA-3′ | 5′-GCCTCTCGAAGAACAAGTCC-3′ |
| *ATG10* | Sl09g047840 | 5′-GGAGAACCCTTGGCAATAGA-3′ | 5′-TAGTCCCACATGGATGCAAT-3′ |
| *ATG12* | Sl12g049310 | 5′-GTGTATGTCAACAGCGCCTT-3′ | 5′-AAACAACCAGGAGTTCTCAGC-3′ |
| *ATG13a* | Sl03g096790 | 5′-GATGTCGACACTTCCGATTC-3′ | 5′-TTGCAGATCCCTGAGAAGAG-3′ |
| *ATG13b* | Sl06g072980 | 5′-CTGTAGGTGCCCTTGTTCAC-3′ | 5′-AAGCTTTGAGCTCCTCCAAT-3′ |
| *ATG18a* | Sl08g006010 | 5′-CAGCGAGTTCACCACTATCC-3′ | 5′-TCCATCCAAGCCAAGAATTA-3′ |
| *ATG18b* | Sl07g006120 | 5′-TTGAGGAGACAGCAACACCT-3′ | 5′-TGTTCTGATGGTTGACGTTG-3′ |
| *NRT1.1* | Sl08g078950 | 5′-CACATCGGAAAATTCGAGATCC-3′ | 5′-GCCGCAATCATAGCTATAATCG-3′ |
| *NRT2.1* | Sl06g074990 | 5′-CAAAGAATTGAAGGATCACCGG-3′ | 5′-AGTGAAAAAGGAGATCCAGGAG-3′ |
| *NR* | Sl11g013810 | 5′-ACGAGGATGACGATGACGAA-3′ | 5′-GCGAGATAAAGGTGGTTCGG-3′ |
| *NiR1* | Sl01g108630 | 5′-GTTAGGCTCAAGTGGCTTGG-3′ | 5′-CAGCACATCCTTCCTCTCCA-3′ |
| *NiR2* | Sl10g050890 | 5′-CATCCGCAGAAACAGGAAGG-3′ | 5′-CCGGAGTTCTCCTGAACCAT-3′ |
| *Ubi3* | Sl01g056940 | 5′-TCGTAAGGAGTGCCCTAATGCTG-3′ | 5′-CAATCGCCTCCAGCCTTGTTGTA-3′ |
| *Actin* | Sl03g078400 | 5′-CTCAGTCAGGAGAACAGGGT-3′ | 5′-GCCTCCAGCCTTGTTGTAAA-3′ |
