# Supplementary material for: Autophagic pathway contributes to low-nitrogen tolerance by optimizing nitrogen uptake and utilization in tomato
Source: Hortic Res. 2022 Mar 23;9:uhac068. doi: 10.1093/hr/uhac068 (PMC9164271; doi:10.1093/hr/uhac068)
Supplement: Web_Material_uhac068 [file web_material_uhac068.zip › 20220216 ___ CN__ __.pdf]

Supplemental Figure S1

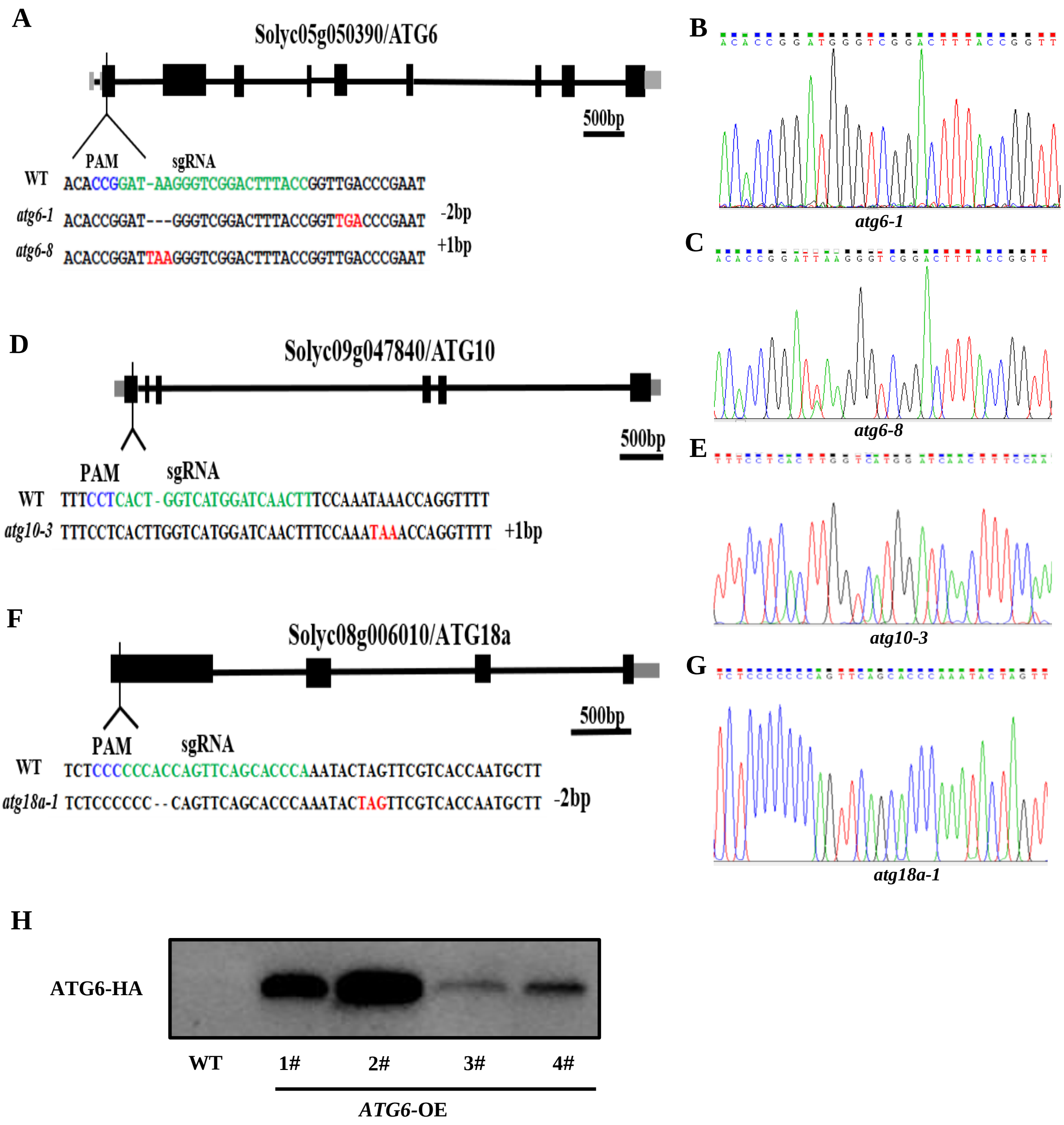

Figure S1. Identification of *atg6*, *atg10* and *atg18a* mutants and two lines of *ATG6* overexpressing plants (OE1 and OE2). DNA sequence comparison of wild-type (WT), *atg6-1* (A), *atg6-8* (A), *atg10-3* (D) and *atg18a-1* (F). Sanger sequencing chromatogram analysis show *atg6-1* (B) and *atg6-8* (C) mutants contain a 2 bp deletion and an 1 bp insertion in the exon of *ATG6* gene, respectively. (E) *atg10-3* mutant contains an 1 bp deletion in the exon on of *ATG10* gene. (G) *atg18a-1* mutant contains a 2 bp deletion in the exon of *ATG18a* gene. Protospacer adjacent motif (PAM) was marked in blue, sgRNA was marked in green and stop codon was marked in red. (H) Western blotting analysis of transgenic plants expressing the HA-tagged *ATG6*-OE plants.

Supplemental Figure S2

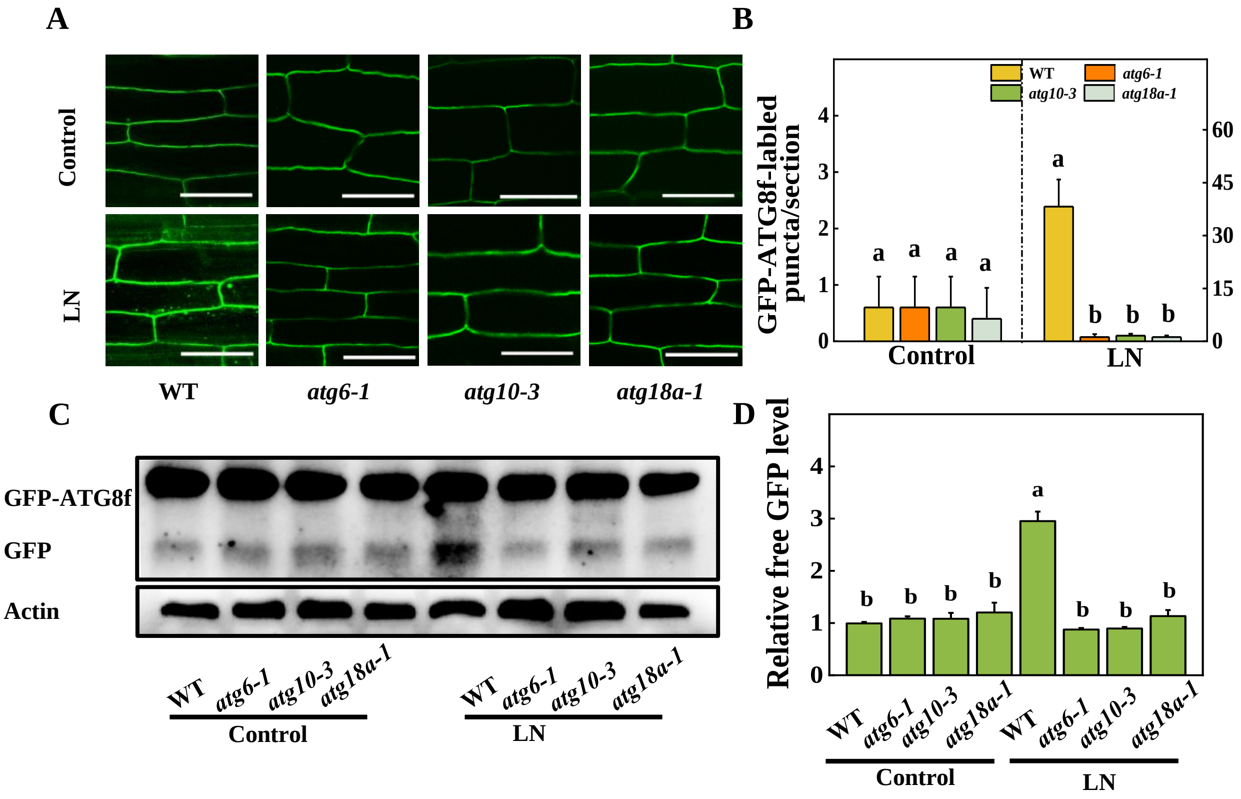

Figure S2. The autophagic acitivities in the roots of WT, *atg6-1*, *atg10-3* and *atg18a-1* mutants under low-N (LN) stress. (A) The direct fluorescence of GFP-ATG8f was detected in the roots of WT and *atg* mutants under LN stress by confocal microscopy. Bars: 25 µm. (B) The number of GFP-ATG8f-labeled puncta per image in (A). (C) The accumulation of GFP-ATG8f proteins in *GFP-ATG8f* overexpressing roots of WT and *atg* plants on the 5th day under LN stress. GFP-ATG8f fusion and free GFP are indicated on the left. (D) Relative free GFP levels in (C), the ratio of free GFP and Actin in control WT was set to 1. Actin was used as a loading control for the western blotting analysis. More than 20 images were measured. All experiments were repeated 3 times with similar results. Different letters indicate significantly differ at $P < 0.05$ according to the Turkey's test.

Supplemental Figure S3

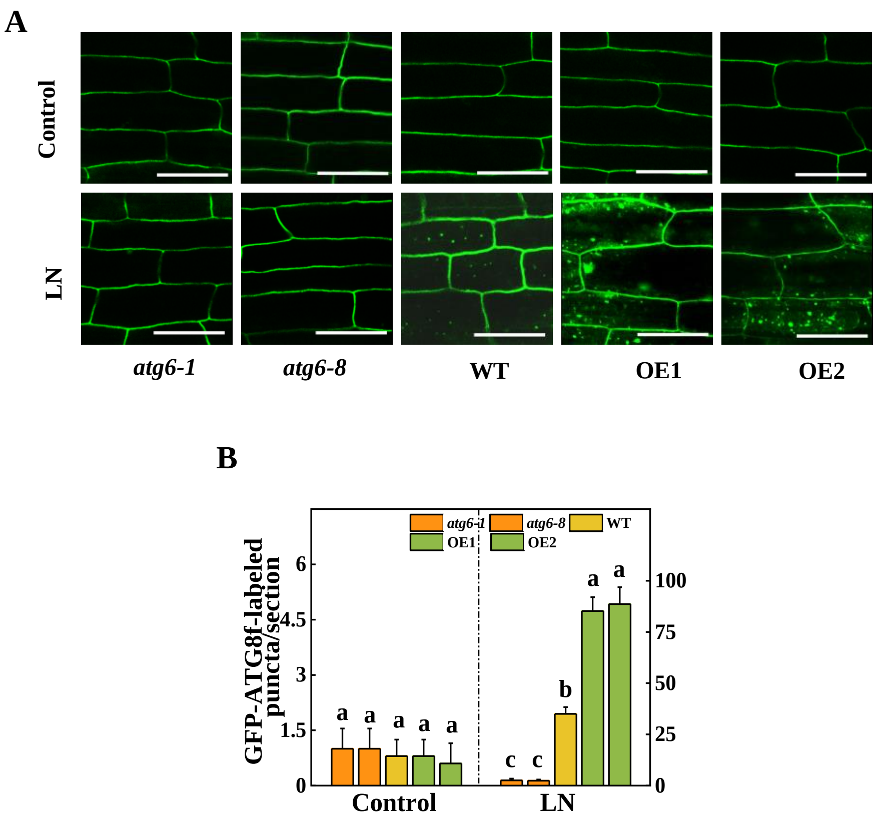

Figure S3. The accumulation of autophagosomes in the roots of *atg6-1*, *atg6-8*, WT and two lines of *ATG6*-overexpressing (OE1 and OE2) plants under low-N (LN) stress. (A) The direct fluorescence of GFP-ATG8f was detected in the roots of *atg6-1*, *atg6-8*, WT, OE1 and OE2 plants under LN treatment by confocal microscopy. Bars: 25 µm. (B) The number of GFP-ATG8f-labeled puncta per image in (A). More than 20 images were measured. All experiments were repeated 3 times with similar results. Different letters indicate significantly differ at $P < 0.05$ according to the Turkey's test.

Supplemental Figure S4

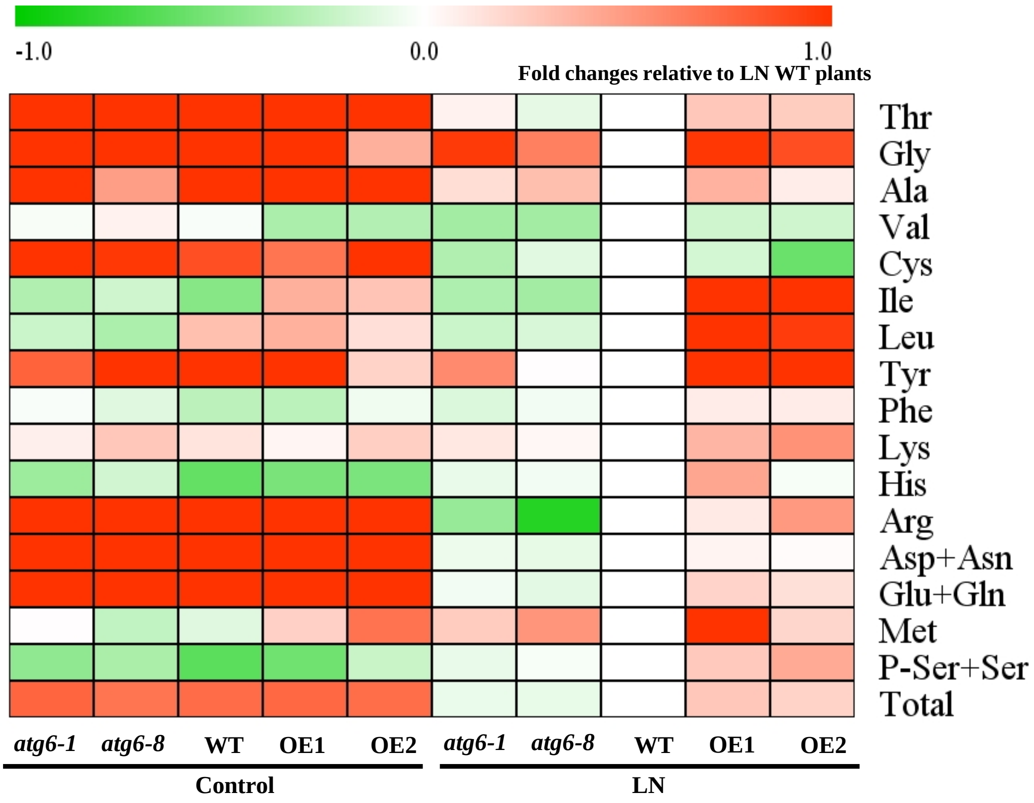

Figure S4. The relative changes of free amino acids in tomato leaves. Heat-map of relative changes of free amino acids in the leaves of *atg6-1*, *atg6-8*, WT and two lines of *ATG6*-overexpressing (OE1 and OE2) plants on the 3rd day under low-N (LN) stress. All values are fold changes of the data detected in LN WT plants; and values of LN WT were set to 0 for intuitive visualization. Decreases and increases are shown in green and red which was drawn by MeV version 4.9. Data represent the mean of 3 biological replicates (± SD).

Supplemental Figure S5

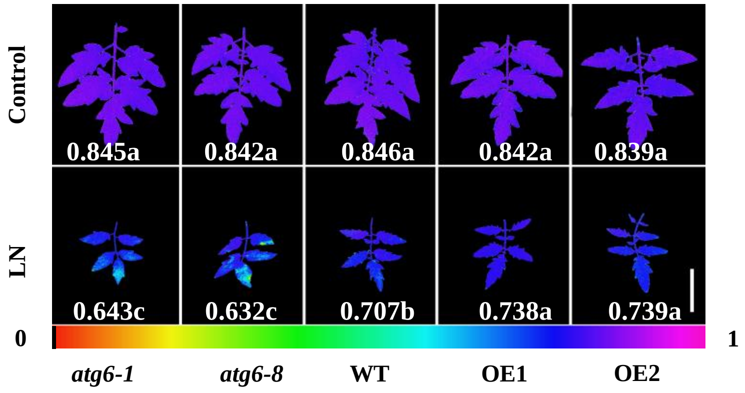

Figure S5. The maximal photochemical efficiency of PSII (Fv/F_m) in *atg6-1*, *atg6-8*, WT and two lines of *ATG6*-overexpressing (OE1 and OE2) plants on the 14th day under low-N (LN) stress. More than 20 images were measured. The experiments with the same experimental design were repeated 3 times with similar results, and representative data from one experiment are shown.

Supplemental Figure S6

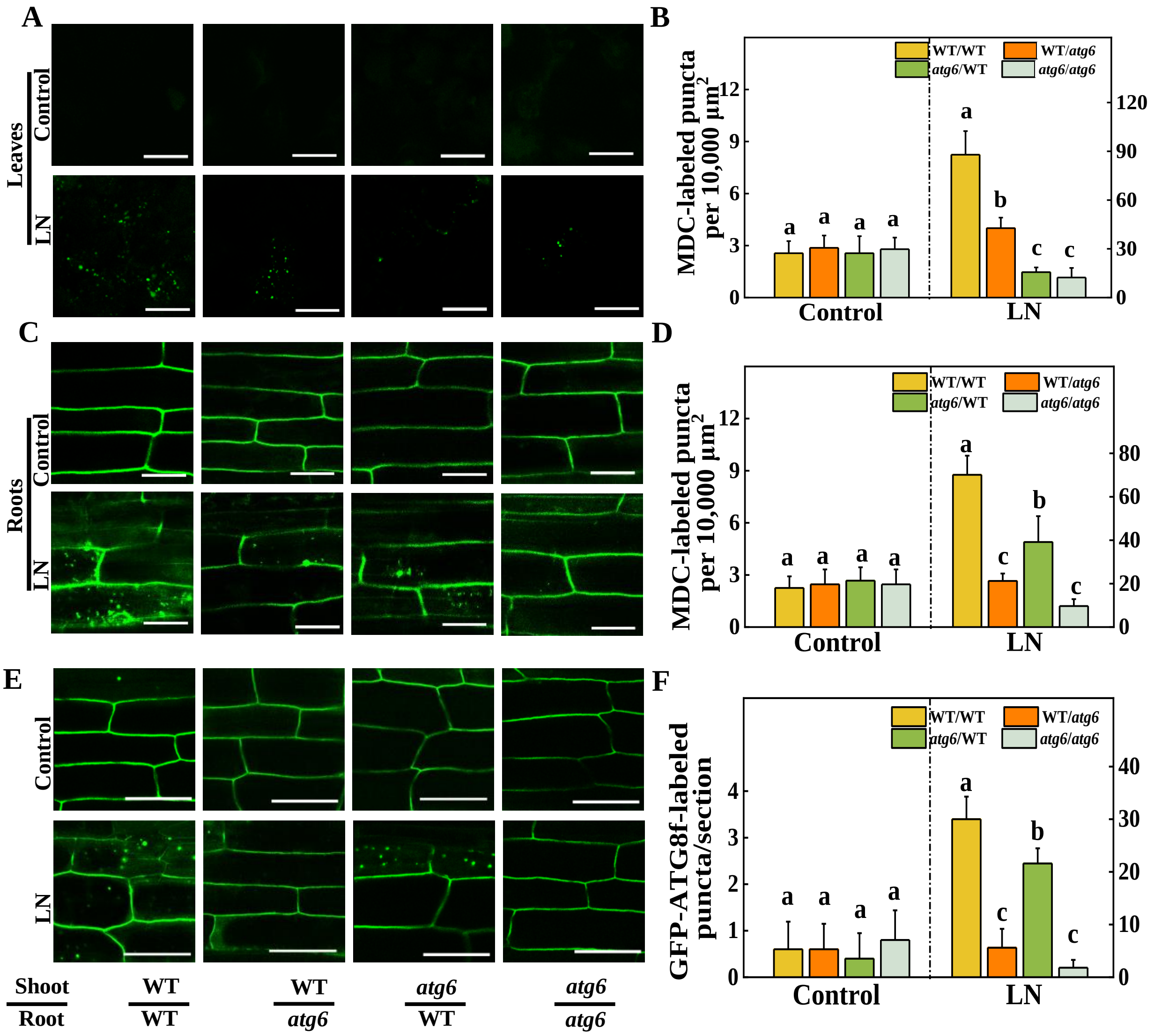

Figure S6. Autophagic activity in grafted plants under low-N (LN) stress. MDC-stained autophagosomes were detected in the leaves (A) and roots (C) of grafted plants on the 5th day under LN stress by confocal microscopy. MDC-stained autophagosomes were shown as green signals. Bars: 25 µm. The number of MDC-stained autophagosomes per image in the leaves (B) and roots (D) was quantified to calculate the autophagic activity, respectively. More than 20 images for each treatment were used for the quantification. (E) The direct fluorescence of GFP-ATG8f was detected in the roots of grafted plants under LN treatment by confocal microscopy. Bars: 25 µm. (F) The number of GFP-ATG8f-labeled puncta per image in (E). More than 20 images were measured. All experiments were repeated 3 times with similar results. For each panel separated by dotted lines, different letters indicate significantly differ at $P < 0.05$ according to the Turkey's test.

Supplemental Figure S7

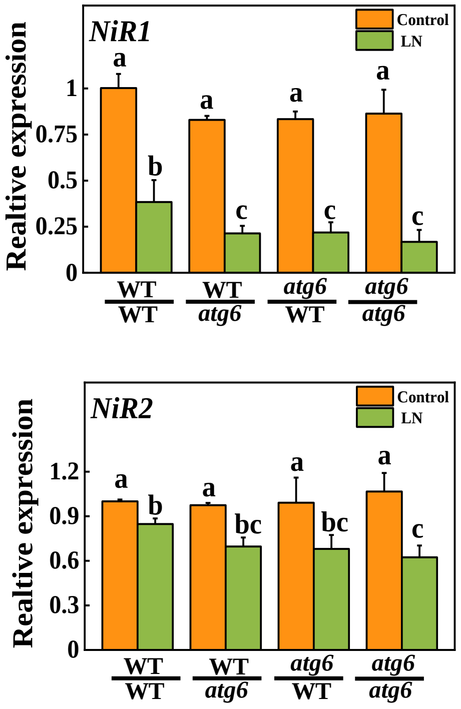

Figure S7. The expression of *NiR1* and *NiR2* in response to low-N (LN) stress. Data represent the mean of 3 biological replicates (± SD). Different letters indicate significantly differ at $P < 0.05$ according to the Turkey's test.
